# An Integrative Network Modeling Approach to T CD4 Cell Activation

**DOI:** 10.3389/fphys.2020.00380

**Published:** 2020-04-23

**Authors:** David Martínez-Méndez, Carlos Villarreal, Luis Mendoza, Leonor Huerta

**Affiliations:** ^1^Instituto de Investigaciones Biomédicas, Universidad Nacional Autónoma de México, Mexico City, Mexico; ^2^Instituto de Física, Universidad Nacional Autónoma de México, Mexico City, Mexico; ^3^Centro de Ciencias de la Complejidad, Universidad Nacional Autónoma de México, Mexico City, Mexico

**Keywords:** T CD4 cells, TCR, Boolean model, complex network, CD28, NDRG1, CTLA-4, AMPK

## Abstract

The adaptive immune response is initiated by the interaction of the T cell antigen receptor/CD3 complex (TCR) with a cognate peptide bound to a MHC molecule. This interaction, along with the activity of co-stimulatory molecules and cytokines in the microenvironment, enables cells to proliferate and produce soluble factors that stimulate other branches of the immune response for inactivation of infectious agents. The intracellular activation signals are reinforced, amplified and diversified by a complex network of biochemical interactions, and includes the activity of molecules that modulate the activation process and stimulate the metabolic changes necessary for fulfilling the cell energy demands. We present an approach to the analysis of the main early signaling events of T cell activation by proposing a concise 46-node hybrid Boolean model of the main steps of TCR and CD28 downstream signaling, encompassing the activity of the anergy factor Ndrg1, modulation of activation by CTLA-4, and the activity of the nutrient sensor AMPK as intrinsic players of the activation process. The model generates stable states that reflect the overcoming of activation signals and induction of anergy by the expression of Ndrg1 in the absence of co-stimulation. The model also includes the induction of CTLA-4 upon activation and its competition with CD28 for binding to the co-stimulatory CD80/86 molecules, leading to stable states that reflect the activation arrest. Furthermore, the model integrates the activity of AMPK to the general pathways driving differentiation to functional cell subsets (Th1, Th2, Th17, and Treg). Thus, the network topology incorporates basic mechanism associated to activation, regulation and induction of effector cell phenotypes. The model puts forth a conceptual framework for the integration of functionally relevant processes in the analysis of the T CD4 cell function.

## 1. Introduction

Antigen-driven T cell responses are performed by the combined activity of many intracellular reactions acting in concerted cascades to reinforce, amplify, diversify and regulate the initial antigenic signal. These processes lead to the clonal expansion and differentiation of the T cells into a variety of effector phenotypes. The extensive availability of biochemical mediators of the signaling network pathways includes cell-specific molecules as well as molecules from the microenvironment. The current model of the early events involved in activation of naive T CD4 cells by antigen-presenting cells (APC) includes three main requirements for completion of the response: (1) the interaction of a cognate peptide bound to a major histocompatibility complex (MHC) molecule with the T cell receptor (TCR-CD3-ζ chain complex, or TCR), along with the binding of the CD4 co-receptor to the MHC molecule, (2) the binding of co-stimulatory molecules to receptors on the T cell membrane, mainly CD28, and (3) the action of cytokines from the micro-environment which determine differentiation of activated cells to particular effector phenotypes. In addition, the activation process includes signaling for its own regulation and induction of the metabolic changes necessary to meet cellular energy demands.

Signaling from the TCR and CD28 converge in the activation of the AP-1, NFAT, and NF-κB transcription factors, which are necessary for the expression of IL-2, a cytokine essential for cell survival and proliferation. In the absence of co-stimulation through CD28, incomplete activation leads to anergy. On the other hand, upon efficient activation, signals that regulate the whole processes are also elicited, being those mediated by CTLA-4 the most well-studied.

Previous approaches to mathematical modeling of the early events of T cell activation and differentiation have been undertaken, integrating detailed pathways of activation from the TCR and co-stimulatory molecules (Klamt et al., 2006; Saez-Rodriguez et al., 2007; Rodríguez-Jorge et al., 2019), as well as the regulation of the cell phenotype by cytokines (Naldi et al., 2010; Martinez-Sanchez et al., 2018; Puniya et al., 2018). The model we put forward is based on a network that abridges basic pathways considered in these publications and incorporates other relevant players as intrinsic elements of the signaling initiated by TCR and CD28 stimulation. In particular, we incorporated those elements involved in anergy (Ndrg1), regulation of the ongoing activation process (by CTLA-4 dimers), and metabolic shifts associated to differentiation toward effector phenotypes (via AMPK-mTOR), so that they importantly influence the active or inactive stage of many of the nodes in the network and have differential roles in the generation of effector phenotypes. The elements included in the model and their inner interactions are documented by abundant experimental data. Boolean logic rules allowed modeling of activation and inhibition reactions, which are typical outcomes of biochemical interactions in signaling pathways. In addition, the affinity competition involved in regulation by CTLA-4 by displacement of CD28 from co-stimulatory molecules was modeled using a continuous differential equation, and was incorporated into the Boolean model. This kind of approach allows modeling of the basic interactions and can be used for the analysis of biological circuits without requiring explicit values of physiological parameters. The model is concise and is put forwarded as a framework for the integration of functional events leading to the response of T CD4 cells.

## 2. Methods

### 2.1. Network Inference

**Figure 1:**
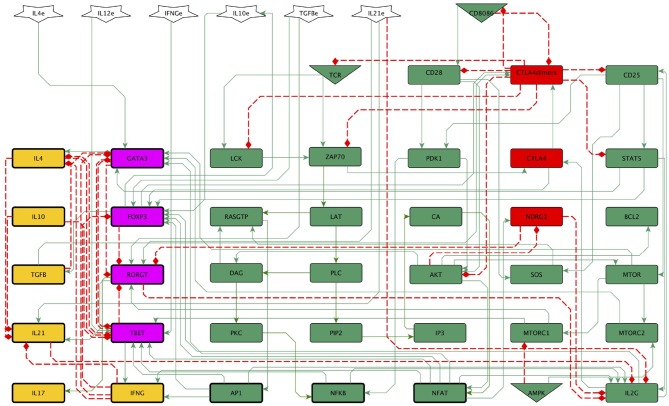
A general 46-node network of the early biochemical interactions induced by TCR activation, co-stimulation, and phenotype-inducing cytokines. The continuous and dotted lines represent activator and inhibitory pathways, respectively. Inputs of the network are the activated TCR, the co-stimulatory molecules CD80/86, the nutrient micro-environment-sensing protein AMPK (triangles), and external cytokines (polygons). The set of functions defining the dynamical system for boolean modeling is shown in Supplementary Table 1. Green and red rectangles represent stimulatory and inhibitory nodes of the activation core, respectively. Purple and yellow rectangles represent phenotype-inducing transcription factors and cytokines produced endogenously, respectively. LAT, LAT-Gads-SLP-76 complex; IL2G, IL2 gene; IL4e, IL2e, IFNγe, IL10e, TGFβe, and IL21e, exogenous cytokines.

**Figure 2:**
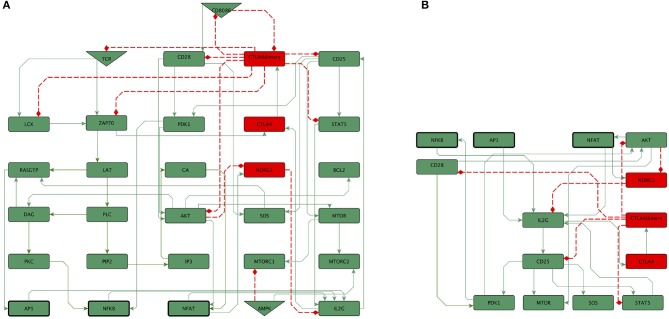
**(A)** The activation core of the network represents downstream signaling induced by TCR and CD28, encompassing the activity of CTLA-4, the anergy factor NDRG1, and the nutrient micro-environment-sensing protein AMPK. The effect of exogenous cytokines is not included. Green and red rectangles represents stimulatory and inhibitory nodes of the activation core, respectively. **(B)** Subnetwork of the core showing the convergence of the activity of NFκB, AP-1 and NFAT for the expression of the IL-2 gene (IL2G). IL2G induces the expression of its own high affinity receptor (CD25). CD25 mediates the activity of PDK1, mTOR, SOS, and STAT5, which positively feedback the expression of IL-2. The anergy-inducing factor Nrdg1 is induced by NFAT and inactivated by Akt. Colors of rectangles correspond to the type of components, as indicated in Figure 1.

**Figure 3:**
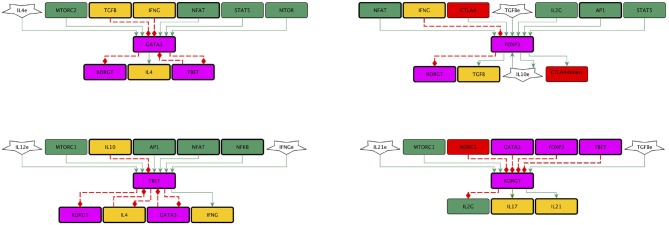
Subnetworks representing the effect of exogenous cytokines on cell differentiation. Exogenous IL-4, IL-2, IFNγ, IL-10, TGF-β, and IL-21 were herein named IL-4e, IL-2e, IFNGe, IL-10e, TGFBe, and IL-21e (polygons). Specific cytokines induce the activity of particular transcription factors (T-bet, GATA3, RORγt, and Foxp3), which guide the differentiation of effector cell subtypes (Th1, Th2, and Treg cells produce IFNγ, IL-4, and TGF-β, respectively, whereas Th17 cells produce IL-21 and IL-17). Cross-inhibitory signals involved in the predominant induction of a cell subtype by particular cytokines are shown. Colors of rectangles correspond to the type of components, as indicated in Figure 1.

The main early events involved in activation and regulation of T CD4 lymphocytes were abridged to construct the 46-node network shown in Figure 1. The network is integrated by several modules, including the one herein called “activation core.” The activation core encompasses downstream signaling from TCR and CD28, and includes the activity of CTLA-4, the anergy factor NDRG1, and the microenvironmental nutrient sensor 5′adenosine monophosphate-activated protein kinase (AMPK) (Figure 2). Four modules associated to the activity of external cytokines guiding differentiation toward four effector phenotypes were added (Figure 3). The set of functions defining the dynamical system for Boolean modeling is shown in Supplementary Table 1. Interactions composing the network were included on the base of experimental information and are outlined below and in Supplementary Table 2, while the continuous modeling involved in co-inhibition by CTLA-4 is described below in the subsection Mathematical modeling.

#### 2.1.1. TCR and CD28 Co-stimulation

We constructed a minimal network encompassing nodes corresponding to key elements elicited by the TCR and CD28 upon interaction with the MHC-peptide complex and co-stimulatory molecules, herein named the network core (Figure 2A). The first defining step produces the phosphorylation of immunoreceptor tyrosine-based activation motifs (ITAMs) in the TCR CD3 molecule by the Lck kinase (Stirnweiss et al., 2013). Phosphorylated ITAMs recruit and activate the tyrosine kinase ZAP-70, which in turn phosphorylates scaffold (LAT and SLP-76) and adaptor (Gads) proteins to form the LAT-Gads-SLP-76 complex. Activation of ZAP-70 also induces the recruitment and activation of the PI3-kinase enzyme (PI3K). PI3k induces in turn phosphorylation and activation of the phospholipase C γ (PLC-γ), which hydrolyses the phosphatidylinositol 4,5-biphosphate (PIP2) to produce the second messengers diacylglycerol (DAG) and inositol trisphosphate (IP3). These molecules stimulate three important branches: promotion of Ca^2+^ entry, activation of Ras, and activation of the protein kinase C-θ (PKC-Cθ). IP3 elicits the release of Ca^2+^ from the endoplasmic reticulum and the entry of extracellular Ca^2+^ into cells through channels located at the cell membrane, leading to the activation of the phosphatase calcineurin which in turn dephosphorylates NFATs, promoting their translocation to the nuclei. DAG diffuses in the plasma membrane and activates and recruits the Ras guanyl-releasing protein (RasGRP) to allow the activation of Ras to form RasGTP. DAG also recruits PKC-θ to the membrane, where it initiates a series of steps promoting the release and entry of active NF-κB into the nucleus (Gaud et al., 2018). Activation of Ras is also performed by the guanine-exchange factor SOS, which is recruited to the plasma membrane to bind LAT through the adaptor protein Grb2, forming a second LAT three-protein complex (LAT-Grb2-SOS). Active SOS is induced through CD28 and CD25 downstream pathways. In the model we considered the requirement of signals from both the TCR (though a pathway involving LAT, PLCγ and DAG) and CD28 signaling (by activation of SOS) for generation of RasGTP, as it has been documented experimentally (Su et al., 1994; Macián et al., 2002; Perez de Castro et al., 2004; Mor et al., 2007; Janardhan et al., 2011). RasGTP initiates a sequence of phosphorylation reactions leading to the activation of the mitogenic protein kinase (MAPK) pathway, which ultimately ends in the formation of AP-1.

Co-stimulation from CD28 enhances TCR signaling and contributes to cell survival and cell cycle progression. CD28 activates PI3K to generate the membrane phosphoinositide PIP3, which recruits Akt and the PDK1 kinase; this conjunction allows the phosphorylation of Akt and the stimulation of downstream pathways that contribute to the activation of NFAT, NF-κB, and AP-1 (Chen and Flies, 2013). Akt can also be activated in a PI3K independent manner through phosphorylation induced by Src SH3 domains (Jiang and Qiu, 2003). The two pathways leading to Akt activation have been introduced in the network (Figure 2B) (Frauwirth and Thompson, 2002; So and Croft, 2013; Jung et al., 2018).

Activation of the AP-1, NFAT and NF-κB transcription factors is necessary for the expression of the IL-2 gene (Figure 2, right). IL-2 induces the expression of its own high affinity receptor subunit, CD25, on the cell membrane (Sereti et al., 2000). In the network, CD25 mediates downstream pathways induced by IL-2, i.e., the activities of PDK1 (Park et al., 2009), SOS (Gadina et al., 1999), the mTORC complex (Marzec et al., 2008), the transcription factors STAT5 (Mahmud et al., 2013), and FoxP3 (Waters et al., 2018). Feedback loops for IL-2 synthesis are thus induced, since the activity of PDK1 ultimately ends in NF-κB and Akt activation, while SOS and STAT5 lead to AP-1 activity. On the other hand, induction of mTOR activity supports the upregulation of metabolism for cell proliferation.

#### 2.1.2. Anergy

It is widely recognized that the lack of co-stimulatory signals from CD28 induces T cell anergy upon TCR-peptide-MHC interaction, highlighting the essential role of co-stimulation. Anergy of T cells is characterized by poor cell proliferation and scarce production of IL-2 (Michel et al., 2000; Kovacs et al., 2005; Round et al., 2005; Schneider et al., 2008b). The N-myc downstream regulated 1 (Ndrg1) protein is a metastasis suppressor that has been previously associated to hypoxia (Sibold et al., 2007) and endoplasmic reticulum stress (Merlot et al., 2019) in non-lymphoid cells. Recently, it was described as a T-cell clonal anergy factor (Oh et al., 2015). It is thought that the calcium-calcineurine-NFAT signaling pathway induces the expression of Ndrg1 via Egr2, whereas signaling from CD28 promotes its inactivation trough phosphorylation by Akt, a major signaling molecule downstream of CD28. When anergic cells are treated with IL-2, Akt becomes active and Ndrg1 is phosphorylated and degraded (Oh et al., 2015). This regulatory circuit of T CD4 cell activation, in which signaling from the TCR induces Ndrg1, whereas signaling from CD28 leads to its inactivation, was incorporated to the activation core (Figures 1, 2).

#### 2.1.3. Co-inhibition by CTLA-4

Once initiated, activation is thoroughly regulated by the activity of the cytotoxic T lymphocyte antigen-4 (CTLA-4), a molecule that is known to be a critical inhibitory “checkpoint” for T-cell activation, thus restricting the immune response. Expression of CTLA-4 is upregulated upon TCR activation and IL-2 production (Perez et al., 1997; Wang et al., 2001), followed by its dimerization and expression at the cell membrane (Darlington et al., 2005). Interaction of CTLA-4 dimers with CD80/86 generates strong inhibitory signals at multiple levels of the activation cascade (Darlington et al., 2005). The affinity of CTLA-4 dimers for CD80/86 is higher than for CD28, thus establishing a competition mechanism (Schneider et al., 2008a). The model also incorporates the observation that expression of CTLA-4 dimers is supported by the transcription factor Foxp3, in agreement with its role in the induction of Treg cells (Wing et al., 2008; Walker, 2013). The interaction of CTLA-4 dimers (CTLA-4dimers) with CD80/86 induces multiple downstream inhibitory signals (Chikuma, 2017). The interaction of CD80/86 with CD28 or CTLA-4 dimers was modeled as a continuous function that satisfies a logistic differential equation. This equation describes the affinity shift of CD80/86 from CD28 toward CTLA-4 dimers (see below).

#### 2.1.4. Differentiation-Inducing Cytokines

The network incorporated the effect of cytokines released by APC and other cells in the microenvironment. They activate intracellular signaling pathways that stimulate or inhibit particular transcription factors for differentiation of effector phenotypes. Th1 cells are induced by IL-12 and IFNγ, express the T-bet transcription factor and produce IFNγ. Th2 cells require IL-4 and are stabilized by IL-2, express GATA3 and produce IL-4, IL-5, and IL-13. Th17 cells require TGF-β and IL-6, IL-21, or IL-23, express RORγt and produce IL-21, IL-17A, and IL-17F. Treg cells require TGF-β and IL-2, express Foxp3, TGF-β and IL-10 in some cases (Hori et al., 2003; Davidson et al., 2007; Zheng et al., 2007; Chen and Flies, 2013). The network included the influence of components of the network core and cross-inhibitory signals involved in the predominant induction of a cell subtype by particular cytokines, as documented in the experimental literature: inhibition of T-bet expression by IL-10; inhibition of GATA3 expression by IFNγ and TGF-β; inhibition of RORγt expression by T-bet and INFγ, Foxp3, GATA3 and the anergy factor NDRG1, and inhibition of Foxp3 expression by IFNγ (Ichiyama et al., 2008; MacIver et al., 2013; Wan, 2014) (Figure 3).

#### 2.1.5. Metabolism in Cell Differentiation

The capacity of T cells to proliferate and adopt effector functions is closely linked to a switch to glycolysis to support increased anabolic activities. It is known that CD28 enhances the levels of Akt activation induced by the TCR signaling. The PI3k-Akt pathway then promotes the activity of the mTORC1 complex, which in turn induces the expression of the hypoxia-inducible factor for promotion of glycolysis, supporting differentiation of naive cells toward the Th1 and Th17 subsets (Dang et al., 2011; Shi et al., 2011). Alternatively, restriction of glucose and essential amino acids can lead to the activation of the nutrient sensor AMPK (MacIver et al., 2013; Man and Kallies, 2015). Active AMPK impairs mTORC1 activity and favors the mTORC2 pathway, leading to a lower energy consumption and supporting differentiation toward the Treg and Th2 subsets (Delgoffe et al., 2011; Michalek et al., 2011; Klysz et al., 2015). The network posited here incorporates these pathways of metabolic regulation as part of the activation core (Figure 2).

#### 2.1.6. Mathematical Modeling

Based on the molecular information described above we reconstructed a regulatory network of 46 nodes for the activation of T CD4 cells mediated by the TCR and costimulation (Figure 1). The network is composed by a central activation core (30 nodes) associated to TCR and CD28 priming (Figure 2), and four differentiation modules associated to the effect of exogenous cytokines conducing to Th1, Th2, Th17, and Treg phenotypes (Figure 3). The regulatory network was converted into a hybrid dynamical system mainly constituted by discrete Boolean variables, but also including a component with a continuous dynamics to simulate the time-dependent affinity shift involved in the interaction of CD80/86 with CD28 or CTLA-4 dimers. This variable, denoted as *CD*8086(*t*), is introduced in the Boolean framework by assuming that it acquires an active or inactive state if its expression level at a time *t* is greater or smaller than a given threshold, similarly as in the hybrid approach introduced by Glass to study regulatory networks with a piece-wise continuous dynamics (Chaves et al., 2006). The set of Boolean functions that defines the dynamical system is shown in the Supplementary Table 1 and the model can be found in SBML-qual format in https://github.com/DrMartinezM/Frontiers-Project.git.

In the Boolean segment of the model each discrete node is described by a variable *x*_*i*_ that takes the value 0 or 1 to represent an inactive (unexpressed) or active (expressed) state, respectively, while continuous variables may acquire any real value in the interval [0, 1]. The specific value of *x*_*i*_ is determined by the discrete mapping *x*_*i*_(*t* + 1) = *F*_*i*_(*x*_1_(*t*), …, *x*_*n*_(*t*)), where {*x*_1_(*t*), …, *x*_*n*_(*t*)} is the set of all regulators of *x*_*i*_ and *F*_*i*_ is a logical function, specific for each node, expressing the relationship between that node and its regulatory interactions. Then, the state of the network is identified with the set of values of all nodes at a given time, {*x*_*i*_(*t*)}. A trajectory is the temporal evolution of the network, described in the synchronous framework by *x*_*i*_(*t*), *x*_*i*_(*t* + 1), *x*_*i*_(*t* + 2) *x*_*i*_(*t* + *n*). The iterative procedure goes on until eventually reaching a steady state, or attractor, determined by the fixed-point condition *x*_*i*_(*t* + 1) = *x*_*i*_(*t*). A different class of steady state is the periodic attractor, defined by *x*_*i*_(*t* + *N*) = *x*_*i*_(*t*), so that the system returns to a given state *x*_*i*_(*t*) after *N* iteration steps. In any case, the steady state condition may be reexpressed in the form *x*_*i*_ = *F*_*i*_(*x*_1_, …, *x*_*n*_) (for fixed *t*), which represents a set of Boolean algebraic equations. The solutions of this equation system yield the entire set of attractors, which may be obtained by means of either numerical or analytical methods. In a first approach, we analyzed the trajectories of the network starting from all possible initial states of the network (Ω = 2^46^) to predict all the attractors of the model with the use of the R package BoolNet using synchronous updating (Müssel et al., 2010). The complete set of attractors was of the fixed-point class. In order to gain further insight on the relevance of each node on the activation, regulation, and differentiation processes we also employed methods of Boolean algebra to derive analytical formulas for the expression of every node of the system (briefly described in the Appendix). The results are presented in Tables 1, 2.

**Table 1:** Compacted rules obtained through recursive algebraic analysis of the original Boolean interactions implicit in the activation core (in absence of exogenous cytokines).

**Node**	**Interactive rule**	**Steady state**
CD80/86	Input	Input
AMPK	Input	Input
TCR	TCR and not CTLA4dim	TCR
CD28	CD80/86 and not CTLA4dim	CD80/86
CD25	IL2G and not CTLA4dim	TCR and CD28
IL2G	NFAT and AP1 and not NDRG1	TCR and CD28
MTOR	CD25 or AKT	CD28
CTLA4	ZAP70 and IL2G	TCR and CD28
CTLA4dim	CTLA4 and not CD8086	0
NDRG1	NFAT and not Akt	TCR and not CD28
MTORC1	MTOR and not AMPK	CD28 and not AMPK
MTORC2	MTOR and AMPK	CD28 and AMPK
AP1	rasGTPr	TCR and CD28
STAT5	CD25 and not CTLA4dim	TCR and CD28
NFAT	Ca or (Ca and Akt)	TCR
NFKB	PKCθ or (PKCθ and PDK1)	TCR
AKT	(CD28 and not CTLA4dim) or PDK1	CD28
BCL2	Akt	CD28
DAG	PLC or (PLC and Akt)	TCR
SOS	CD28 or (CD28 and CD25)	CD28
rasGTPr	LAT and SOS and DAG	TCR and CD28
PDK1	CD25 or CD28	CD28
LCK	TCR and not CTLA4dim	TCR
ZAP70	TCR and LCK and not CTLA4dim	TCR
LAT	ZAP70	TCR
PLC	ZAP70	TCR
PIP2	PLC	TCR
IP3	PIP2	TCR
Ca	IP3	TCR
PKCθ	DAG	TCR

*The activation state of each node in the left column is obtained by introducing explicit values (0 or 1) for the elements in the central or right columns and use of the rules for the logical connectives and, or, not. The central column corresponds to an intermediate evaluation step leading to simplified rules for the upper 12 nodes employed in the analysis of attraction basins. The right column corresponds to the final evaluation step defining all possible steady states. It is observed that the expression value of each node in the left column is uniquely defined by TCR, CD28 (determined by CD80/86), and AMPK*.

**Table 2:** Compacted rules and steady states associated to differentiation to functional cell subsets: Th1, Th2, Treg, and Th17.

**Node**	**Interactive rule**	**Steady state**
		Th1
IL12e	Input	1
IFNGe	Input	1
TBET	MTORC1 and NFKB and NFAT and AP1	TCR and CD28 and not AMPK
	and IL12e and IFNGe	
IFNG	TBET and AP1 and NFAT	TCR and CD28 and not AMPK
		Th2
IL4e	Input	1
GATA3	MTORC2 and STAT5 and NFAT and IL4e	TCR and CD28 and AMPK
IL4	GATA3	TCR and CD28 and AMPK
		Treg
TGFBe	Input	1
IL10e	Input	1
FOXP3	(AMPK and NFAT and STAT5 and AP1	TCR and CD28 and AMPK
	and IL2G and TGFBe and IL10e) or	
	(IL10 and TGFBe and IL10e and CTLA4)	
	or (TGFB and TGFBe)	
IL10	FOXP3 and TGFBe	TCR and CD28 and AMPK
TGFB	FOXP3	TCR and CD28 and AMPK
		Th17
TGFBe	Input	1
IL21e	Input	1
RORGT	CD28 and MTORC1 and IL12e and TGFBe	TCR and CD28 an not AMPK
IL21	IL21e or RORGT	TCR and CD28 an not AMPK
IL17	RORGT	TCR and CD28 and not AMPK

*In each case, it has been assumed that exogenous cytokines defining a specific microenvironment are expressed, while the rest is absent. The respective microenvironments correspond to IL12e and IFNGe (Th1), IL4e (Th2), TGFBe and IL10e (Treg), and TGFBe and IL21e (Th17). Besides exogenous cytokine expression, each steady state requires the activation of TCR and CD28, in other words, requires the expression of the activation core. AMPK acts like a switch promoting either cytotoxic or inflammatory immune responses (Th1 or Th17), or alternatively, humoral or regulatory responses (Th2 or Treg)*.

In contrast with the synchronous scheme, an asynchronous iterative approach may be also considered: *x*_*i*_(*t* + τ_*i*_) = *F*_*i*_(*x*_1_(*t*), …, *x*_*n*_(*t*)) where τ_*i*_ is a characteristic updating time for each node. In this case, the fixed-point condition *x*_*i*_(*t* + τ_*i*_) = *x*_*i*_(*t*) leads to exactly the same set of algebraic Boolean equations obtained in the synchronous method, and thus, the sets of fixed-point attractors coincide (Chaves et al., 2006). However, the size of the attraction basins may differ with respect to the synchronous approach. In fact, in the asynchronous framework the size of the basins depends on the different choices of the iteration steps τ_*i*_ (Chaves et al., 2006).

As mentioned before, the translocation of CTLA-4 dimers to the cell membrane and its concomitant interaction with CD80/86 generates an affinity competition with CD28 to form a complex with CD80/86, since the affinity of CTLA-4 dimers for this molecule is higher than the initially engaged activator CD28. To describe this time-dependent process we assumed that the capacity of CD80/86 to form a complex with CD28 was characterized by a continuous function *CD*8086(*t*). On the other hand, the linkage of CD80/86 with CTLA-4 dimers was accounted by a negative correlation described as 1 − *CD*8086(*t*). On these terms, the dynamics of the stimulatory capacity of CD80/86 was determined by a logistic differential equation involving the affinity competition of CD28 and CTLA-4 dimers:

(1) $$\begin{array}{l} \frac{d}{dt}CD8086\left(t\right)=\beta \;CD8086\left(t\right)\left(1-CD8086\left(t\right)\right), \end{array}$$

where the parameter β is a reaction rate. The solution of Equation (1) is

(2) $$\begin{array}{l} CD8086\left(t\right)=\frac{1}{e^{\beta \left(t-t_{d}\right)}+1}, \end{array}$$

where *t*_*d*_ is the time in which the stimulatory capacity of CD80/86 is downregulated to half its initial value, *CD*8086(*t*_*d*_) = 1/2. This function shows a sigmoid structure (see Figure 6), with *CD*8086(*t*) ~ 1, if *t* < *t*_*d*_, and *CD*8086(*t*) ~ 0, if *t* > *t*_*d*_. Clearly, *t*_*d*_ must satisfy the condition *t*_*d*_ ≥ *t*_*ac*_, where *t*_*ac*_ is the time where the activation core is thoroughly expressed. It may be observed that in the limit β ≫ 1, *CD*8086(*t*) becomes a step-like function. As a consequence, the activation state of *CD*8086(*t*) may be considered as a discrete variable whose value depends on a threshold value θ:

$$\text{CD}80/86=\{\begin{array}{cc} 1 & \text{if }CD8086\left(t\right)\geq \theta \text{;}\\ 0 & \text{if }CD8086\left(t\right)<\theta . \end{array}$$

In this work we assumed that β = 50 and θ = 0.5. Accordingly, the activation core and effector phenotypes may remain expressed during a period *T* = *t*_*d*_ − *t*_*ac*_ as far as *CD*8086(*t*) ≥ 0.5. Afterwards, regulation of the T cell activity should conduce to an anergy state. The introduction of a discrete character of CD80/86 makes it possible the use of available standard methods for the analysis of Boolean networks.

Each fixed-point attractor may be reached from a number ω of different initial conditions, where ω denotes the size of the attraction basin. Since the probability that a phenotypic pattern emerges is given by *p* = ω/Ω, it is relevant to evaluate the size of these basins to investigate the probability for the expression, for instance, of an activated or an anergic state. With that purpose, the 30-node network core was analyzed by means of numerical methods to determine the relative size of the attraction basins in the asynchronous approximation. The calculation of these basins involves the evaluation of the number of different paths in which a given attractor is reached from a large set of random initial conditions. It was verified that the resulting relative proportions *p* = ω/Ω were independent of the number of initial conditions. As pointed out above, the use of an asynchronous scheme yields different sizes for the attraction basins as those obtained with synchronous iterations. However, the relative sizes depend on the arbitrary lengths of the iterative steps. Since the results arising in the synchronous approach are consistent with our biological expectations, we preferred to report only results arising in this case to provide a qualitative insight of the activation phenomenology. We consider that a more thorough discussion of this problem should involve a continuous approach.

In order to obtain a graphical representation of the basins of attraction, the 30-node core network was formally reduced by employing the methods of Boolean algebra mentioned above. This method allowed to obtain an equivalent reduced version involving only 12 nodes, which leads to the same set of attractors provided by the original network. The resulting network is described in Table 1. It is important to notice that the reduction method did not discard the inherent action of the nodes eliminated, but incorporated them into a simpler formal scheme that allows to visualize the relative proportion of the size of the attraction basins.

## 3. Results

### 3.1. Network Steady States Correspond to Cellular Functional States

Figure 1 shows the 46-node network integrating inputs (TCR, CD8086, AMPK and exogenous cytokines) with downstream signaling events involved in activation of naive lymphocytes. It incorporates signaling events induced by the TCR and the co-stimulation elicited by the interaction of CD80/86 with CD28, the downstream expression of CTLA-4 with displacement of CD28 from the CD80/86 ligand, the activity of the nutrient sensor AMPK, and the effect of cytokines able to induce differentiation to effector phenotypes.

**Figure 4:**
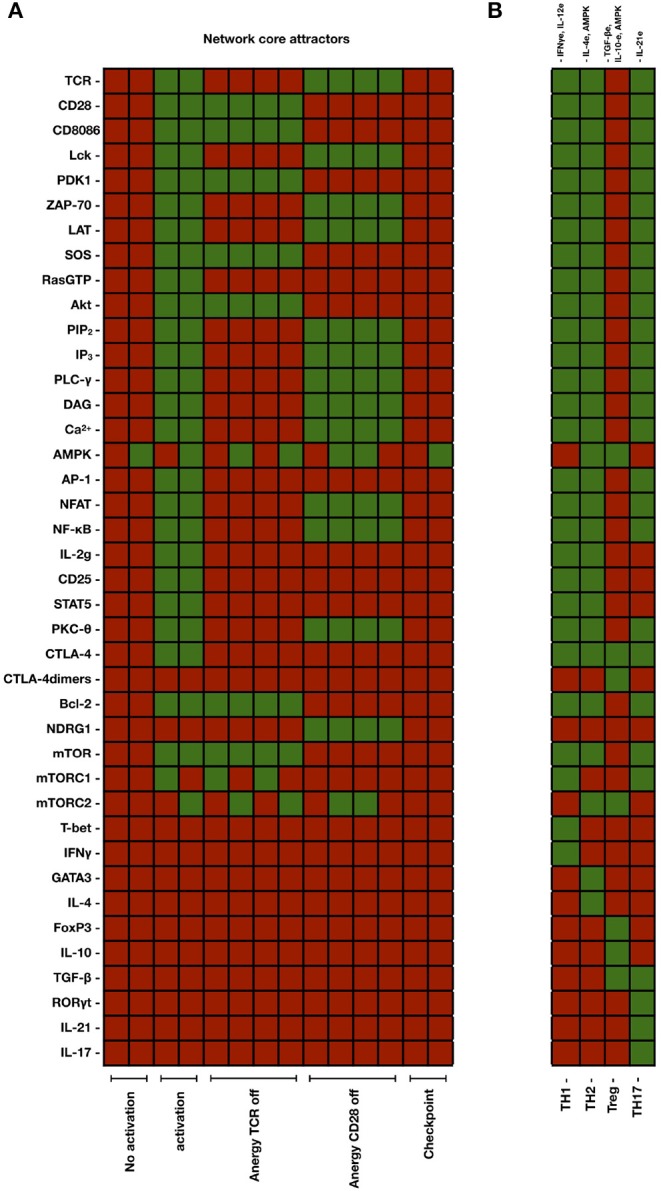
**(A)** Set of attractors generated by the 30-node network corresponding to the activation core and by considering the set of all possible initial conditions (2^30^). Active and inactive nodes are shown in green and red, respectively. Outputs indicating activation were defined as the expression of the IL2G, AP-1, NFAT, and NF-κb transcription factors. The network dynamics generated attractors corresponding to states of no activation, TCR and CD28-induced activation, anergy (when TCR or CD28 were inactive), and immune checkpoint (when CTLA-4 and CTLA-4 dimers are expressed). **(B)** Set of attractors arising from the complete 46-node network (activation core and modules associated to induction of effector phenotypes). In this case, attractors were generated by setting the initial conditions of the activation state (TCR = 1, CD28 = 1, CD80/86L = 0). In addition, the indicated cytokines were set as positive inputs. AMPK was set as active for differentiation to Th2 and Treg cells. Differentiation to effector phenotypes was associated with the production of particular cytokines (INF-γ for Th1, IL-4 for Th2, IL-21 for Th17, and IL-10 and TGF-β for Treg subsets, respectively).

Driven by the network interactions, the dynamical evolution of the system conduces from all possible initial states to a set of 18 final steady states (attractors) presenting particular patterns of active and inactive nodes. These attractors are shown in Figure 4, as well as in Tables 1, 2. The attractors identified with states of activation are thoroughly expressed at time *t* = *t*_*ac*_, whereas the attractors corresponding to the checkpoint are induced at longer times determined by *t*_*d*_. As pointed out in the methodology section, the activation core and effector phenotypes will remain expressed during the time interval *t*_*d*_ − *t*_*ac*_ as far as CD80/86(*t*) > 0.5, when the CD80/86-CD28 interaction is active (Figure 6).

Outputs indicating activation were defined as the expression of the IL-2 gene (IL2G), and AP-1, NFAT and NF-κB transcription factors. Four attractors corresponding to effector phenotypes were associated to the expression of specific transcription factors and production of endogenous cytokines: T-bet and INFγ for Th1, GATA3 and IL-4 for Th2, RORγt and IL-21 for Th17, and Foxp3, IL-10, and TGF-β for Treg subsets, respectively. On these terms, the resulting attractors were classified as follows (numbers in parenthesis indicates the number of attractors consistent with each specific condition): no activation (2), activation (2), anergy associated to inactive TCR (4) or inactive CD28 (4), regulation by CTLA-4 (immune checkpoint) (2), and effector phenotypes Th1 (1), Th2 (1), Th17 (1), and Treg (1). Several attractors exhibited almost identical patterns, except for the activation state of AMPK and CD80/86, which may be either expressed or inhibited. Thus, the two activation attractors resulting from the concurrent priming of TCR and CD28 were associated with the two possible states of AMPK. Likewise, attractors corresponding to the states of no activation and anergy appeared in multiples of 4. This number also reflects the two possible states of the nutrient sensor AMPK and CD80/86. Finally, the two attractors related to the immune checkpoint (which arises with active TCR, CD28, and CD80/86) differ in the expression of AMPK. The gradual shutdown of active nodes due to the interaction of CD80/86 with CTLA-4dim is shown in the following section.

Experimental data indicate that NFAT signaling pathways induce the expression of the Ndrg1 anergy protein via Egr2, whereas signals from CD28 inactivate it via Akt. In the absence of co-stimulation, expression of Ndrg1 provokes a profound lost of the ability of cells to produce IL-2 (Oh et al., 2015). In the model, NFAT induces the activity of Ndrg1, whereas signaling from CD28 inactivate it through the PDK1-Akt pathway (Figure 2B). Thus, when TCR is turned on and CD28 is not active, the activity of Ndrg1 is allowed and the expression of IL-2 is inhibited, leading to anergy (Figure 4). Absence of co-stimulatory signals from CD28 also avoids the activation of PDK1 and SOS. The absence of Akt, PDK1 and SOS ultimately lead to lack of activity of the AP-1 transcription factor, thus reducing the reinforcement of the activation signals elicited by the TCR (Figure 4). The model shows that the NFAT and NFκB transcription factors remain active in the anergic state. This result is due to the fact that these factors are also induced by redundant pathways downstream the TCR, and it can be proposed that their level of activity during the anergic state may depend on the extent of TCR signaling. Thus, the model shows that full activation of the TCR in the absence of CD28 signaling ultimately leads to impaired IL-2 and AP1 expression. This is compatible with a strong inhibitory activity of Ndrg1 on IL-2 production, so that it can overcomes the activation signals induced by the TCR. On the other hand, the model shows that the profile of active nodes in anergic vs. Treg cells is different (Figure 4). This is in agreement with observations based on gene expression analyses comparing anergic and regulatory T cells of the same Ag specificity showing that several genes that are upregulated in activated T cells are altered similarly in anergic cells (Knoechel et al., 2006). These studies and the present model suggest that anergy may reflect a partial activation phenotype but failure to fully commit to effector lineages.

### 3.2. Intermediate States of Activation and Regulation

**Figure 5:**
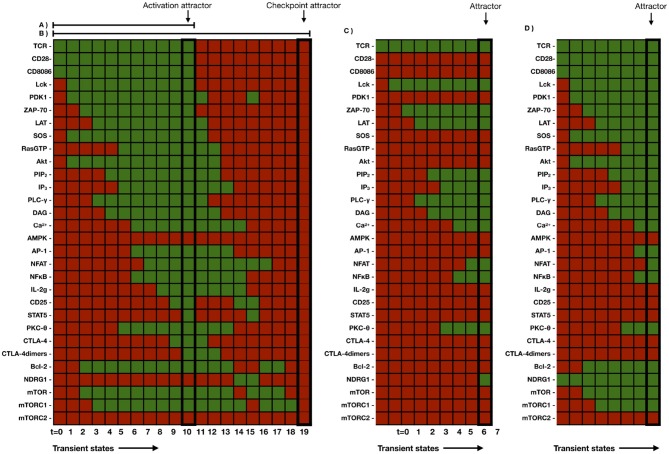
**(A)** Intermediate states leading to T lymphocyte activation and anergy. Active and inactive nodes are shown in green and red, respectively. The transition states from time steps 0 to 10 represent progressive activation. CTLA-4 and CTLA-4 dimers are expressed in a late state of activation (step 10). **(B)** Persistent activation leads to the sustained expression of CTLA-4 dimers; interaction of this molecule with CD80/86 sequentially inactivates multiple nodes (steps 11–18) until the immune checkpoint state is reached. **(C)** Intermediate states leading to the anergy attractor shown in Figure 4 in the absence of co-stimulation. **(D)** Turning on the Ndrg-1 node at the onset of activation through the TCR and CD28 (simulating activation of Ndrg-1-overexpressing cells) leads to partial activation but inhibition of IL-2 synthesis.

Stimulation of the TCR and CD28 in the context of the continuous contact of CD28 with co-stimulatory molecules (CD80/86) leads to the expression of CTLA-4 dimers (CTLA-4dim), which in turn displaces CD28 from the CD80/86 ligands and imparts inhibitory signals over multiple steps in the activation cascade (Vandenborre et al., 1999; Khailaie et al., 2018). CTLA-4 dimers have greater affinity for CD80/86 than CD28 and thus overwhelms the co-stimulatory signal. In the model, CD80/86 binds and activate CTLA-4 dimers. Active CTLA-4 dimers inhibits the form of CD80/86 that binds CD28, thus disabling this interaction. In these conditions, the attractor generated corresponded to a complete inhibition of TCR and CD28 signaling pathways (Figure 4). In the model, intermediate states can be visualized as patterns of transiently activated and inactivated nodes in the route toward the activation and checkpoint steady states. This dynamics was generated by setting the network initial state with TCR and CD28 as active inputs, in the absence or presence of activating CD80/86. Only the network activation module was considered. In these conditions, the system generated intermediate states representing the steps of the activation state and then the sequential inactivation of multiple nodes (Figure 5). Thus, the model reproduced a situation in which TCR activation and costimulation relentlessly quenched the system after the high affinity interaction of CTLA-4 dimers with CD80/86. The attractor corresponding to the immunological checkpoint had the largest attraction basin (see below).

**Figure 6:**
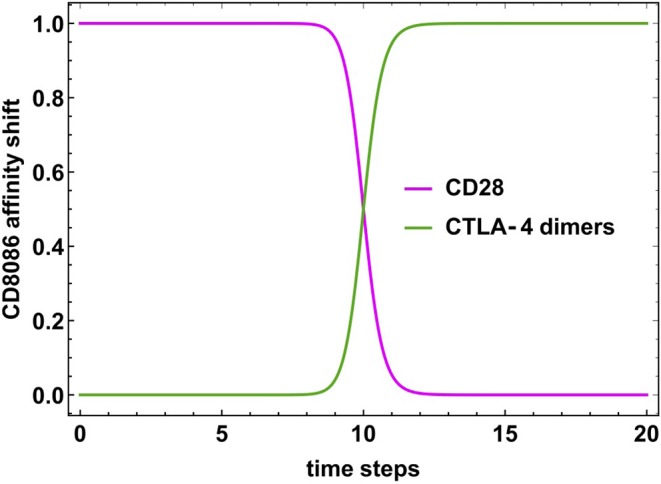
Time-dependent activity of CD80/86 due to the shift of its relative affinity for CD28 and CTLA-4 dimers. The magenta curve describes the linkage strength of the CD80/86-CD28 complex which shows a steep decrease at *t*_*d*_ ≃ 10. The green curve describes the anticorrelated behavior of the linkage strength of CD80/86-CTLA-4 dimers complex.

Figures 5C,D compare the intermediate states leading to the anergy attractor with those observed in conditions of full activation in the presence of Ndrg1 at the initial state. The last condition simulates experiments performed to determine the effect of the overexpression of Ndrg1 at the onset of activation. It can be observed that the full stimulation (through TCR and CD28) of Ndrg-1 over-expressing cells leads to the activation of several nodes downstream TCR and CD28 and to the activity of the AP-1, NFAT and NFκB transcription factors; however, an early inhibition of the expression of the CD25, STAT5 and the IL-2 gene is observed, thus leading to lack of IL-2 synthesis. This result agrees with experimental observations indicating that overexpression of Ndrg1 overcomes the activation signals induced by the TCR and CD28, leading to a partial activation phenotype (Oh et al., 2015).

### 3.3. Basins of Attraction and Model Dynamics

For a particular network, basins of attraction reflect the number of transient states converging to an attractor from all possible initial conditions. Theoretically, large basins of attraction implicate that the network can converge more easily (with less energy cost) toward such attractors than to others with smaller basins. Therefore, large basins of attraction are less prone to be perturbed by changing conditions than small basins. Instead, small basins implicate that conditions to reach the attractor are highly restricted and that convergence to the attractor is more susceptible to changing conditions. In mathematical models of cellular processes, the size of basins gives an estimation of the cell commitment to acquire a given function or phenotype (Demongeot et al., 2010).

The relative size of the basins was the following: 31.26 % was associated with no activation, 6.25% to activation by the TCR and CD28, 9.37% to anergy induced by TCR signaling in the absence of CD28 signaling, 7.03% to anergy due to TCR only, and 46.10% to the checkpoint state. Thus, the largest basins corresponded to no activation and the immune checkpoint, while a small basin corresponded to the activation state. For visualization purposes, a reduced 12-node network was used and graphic representations obtained are shown in Figure 4.

### 3.4. Discussion

In this paper we put forward a concise hybrid Boolean model of the main early signaling events in CD4 T-cell activation. The integrated network constitutes a theoretical framework for organizing and interpreting the vast corp of information on this issue. The mathematical model predicts stable states consistent with functional features of T CD4 lymphocytes: activation under TCR and CD28 stimulation, anergy in the absence of one of these cues, shutdown of the activation cascades by CTLA-4, and differentiation to effector cell phenotypes coupled to corresponding metabolic switches. The model is compatible with the late expression of the inhibitory molecule CTLA-4 in the activation cascade and incorporates the displacement of CD28 from the co-stimulatory ligands. The network topology shows that the expression of every component of the network is exclusively determined by the state of activation of the TCR, CD28, AMPK and the differentiation-inducing cytokines.

The network is based on a central activation module (core) and four sub-networks representing the induction of particular effector cell subtypes by the action of micro environmental cytokines. The core included the function of Ndrg1, CTLA-4, mTOR, and AMPK. Differentiation to effector subtypes was reproduced by incorporating modules representing abridged pathways of the effects of external cytokines on the corresponding transcription factors (TBET, GATA3, FOXP3, and RORgt), as shown in previous reports (Naldi et al., 2010; Mendoza, 2013; Martinez-Sanchez et al., 2018; Puniya et al., 2018). Likewise, activation of transcription factors induced by cytokines are influenced by the activation core through the activity of NFAT, NFkB, AP1, STAT5, mTOR, mTORC1, mTORC2, and CTLA-4, in agreement with the experimental literature. This modular construction allows an efficient study of concurrent pathways and may allow the incorporation of additional components at several levels.

IL-2 is the first cytokine produced upon T cell activation and is essential for survival, proliferation and immune regulation. The core of the model represents the synergistic signaling induced from TCR and CD28 for activation of the AP-1, NFAT, and NFκB transcription factors, which are required for IL-2 synthesis. The network involves a feedback circuit where the activity of the IL-2 gene induces the expression of CD25, which after binding to IL-2, reinforces the expression of the IL-2 gene throughout multiple pathways. The model recovers the manifestation of anergy in the absence of CD28 activity as a partial activated state but no production of IL-2, in agreement with experimental observations (Knoechel et al., 2006; Oh et al., 2015). On the other hand, the model incorporates the activation of the mTORC1 complex as a result of signaling from CD25 and TCR, then inducing differentiation of cells into the Th1 and Th17 subsets. In contrast, activation of AMPK promotes the induction of mTORC2 and antagonizes that of mTORC1, supporting differentiation of cells toward the Treg and Th2 subsets. The model recovers different metabolic profiles associated to these alternative effector functions.

The interaction of CTLA-4 dimers with CD80/86 or the regulatory factors FOXP3 and TGFB conduced to intermediate states representing the sequential inactivation of multiple nodes leading to the activation arrest known as the immune checkpoint. The largest basin of attraction corresponded to the immune checkpoint and the smallest one to activation, which seems consistent with the need to regulate the immune response to avoid deleterious effects arising from over-expression. In the model this is a consequence of the fact that expression of CTLA-4 is necessarily induced after TCR and CD28 activation and inhibits activation at multiple levels. Since the size of basins reflect the theoretical likelihood of the cell commitment to a given function or phenotype, the model indicates that progression of activation, once initiated by TCR and co-stimulation, will be greatly constrained by regulatory mechanisms. Combined with the large basin of attraction of the no activation state, these results may reflect the highly restrictive conditions necessary for initiation and maintenance of T CD4 cell activation.

In the model by Rodríguez-Jorge et al., Lck is activated by CD45, CD4, LCKr, TCR, and CD28. The first three have a basal value 1, while TCR and CD28 are inputs, so that only the activation of these later elements is relevant for Lck expression. Our model does not include an interaction of Lck with CD28. It intrinsically assumes that Lck is already prompted by other elements, and that it is fully activated only after stimulation through the TCR, which is known to stabilize the active form of Lck. The model by Rodríguez-Jorge et al. also includes multiple influences of Fyn on steps proximal to TCR as well as pathways from CD6, which reinforce TCR signaling for the stabilization of the immunological synapse. The contribution of Fyn and CD6 is not explicitly considered in our model, which assumes that TCR is already fully activated. The participation of these elements may be included in a later extension of the model considering the network proposed by these authors. Now, in contrast with the model of Rodríguez-Jorge *et al*., we show that both NFAT and NFκB transcription factors remain active in the anergic state (while AP1 is inhibited), since the activation of NFκB is promoted by PKCθ, whose expression depends on a signaling pathway downstream the TCR, as discussed above. This observation is compatible with gene expression analysis showing that anergic cells reflect a partial activation phenotype (Knoechel et al., 2006). However, this discrepancy could be resolved by experimental studies specifically focused on the role of these molecules during anergy.

Careful scrutiny of the network reveals that the processes that determine T cell activation and differentiation involve self-regulatory processes mediated by the crossed interaction of the activation core with inflammatory and regulatory subnetworks, these latter depending on the expression of specific cytokines and transcription factors (Figures 1–3). Particularly, the role of IL-2 in activation is modulated by a feedback inhibition through the inflammatory factors RORγt and IL-21, while the inhibitory activity of CTLA-4 dimers is promoted by the immune regulation factors TGF-β and FOXP3. On the other hand, the expression of central components, such as PKCθ depend on up-stream signals that include IL-2 and CTLA-4 dimers, thus defining closed regulation loops of the activation and effector modules.

The minimal regulatory network studied in this work considers some of the basic elements included in previous works (Klamt et al., 2006; Saez-Rodriguez et al., 2007; Rodríguez-Jorge et al., 2019), in a abridged form; furthermore, it incorporates other crucial elements involved in the early signaling events leading to CD4 T-cell activation. This model may constitute a conceptual framework to integrate the main early signaling events involved in the T CD4 cell activation through the TCR, co-stimulation and micro-environmental factors, and it can be enriched by the addition of components regulating different elements in the signaling cascades. Likewise, other pathways as those mediating cell adhesion and motility may be incorporated. Furthermore, the dichotomous Boolean approach considered here can be straightforwardly extended to a continuous model in order to incorporate variable strengths of interactions and expression levels of the network components, to provide quantitative descriptions of activation of T CD4 cells as previously applied to modeling of differentiation and plasticity in lymphoid cells (Mendoza, 2013; Mendoza and Méndez, 2015; Martinez-Sanchez et al., 2018;Puniya et al., 2018).

## Data Availability Statement

The datasets generated for this study are available on request to the corresponding author.

## Author Contributions

DM-M, CV, LM, and LH contributed to the conception of the model. DM-M and LH designed the regulatory network. DM-M and CV constructed the logical propositions, conducted the numerical experiments, and performed the analysis of the system dynamics. All authors contributed to the interpretation of results and contributed in writing and review of the manuscript.

## Conflict of Interest

The authors declare that the research was conducted in the absence of any commercial or financial relationships that could be construed as a potential conflict of interest.

## Figures and Tables

**Figure 7:**
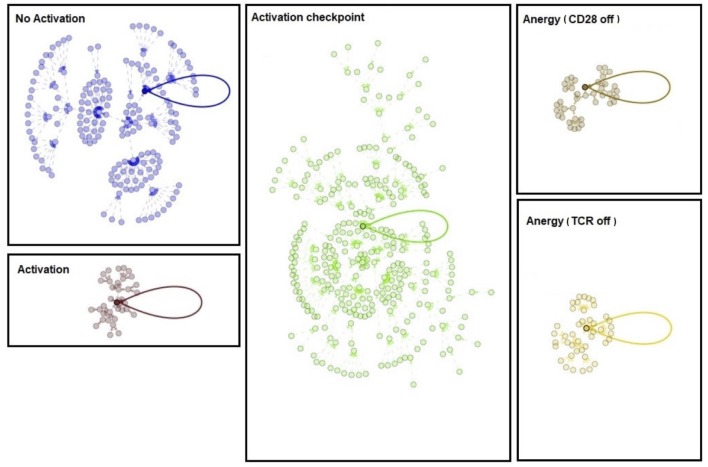
Graphic representations of basins of attraction for the activation core obtained with a simplified 12-node network. The rules involved in the simplified network were rigorously obtained by straightforward use of Boolean algebra as described in the Appendix. In the graphs, initial conditions are represented by the outside layers. From these states, the system transits through intermediate state layers before reaching the final steady state indicated by the loops. The largest basins of attraction are those related with no activation and immune checkpoint states.

## Appendix

In general, the attractors of discrete dynamic mappings are specified by the condition:

$$x_{i}\left(t\right)=x_{i}\left(t+\tau_{i}\right)=F_{i}\left[x_{1}\left(t\right),x_{2}\left(t\right),\ldots ,x_{n}\left(t\right)\right],$$

where τ_*i*_ is the updating time for node *i*. This condition transforms the dynamical mapping into a time-independent set of coupled Boolean algebraic equations *x*_*i*_ = *F*_*i*_[*x*_1_, *x*_2_, …, *x*_*n*_], for fixed *t*, whose solutions yield the whole set of attractors of the system. These solutions are usually obtained by recurring to numerical methods; however, in a number of cases analytical solutions may be obtained by straightforward use of the axioms of Boolean algebra. Denoting the logic connectives as and = ∧, or = ∨, not = ¬, the Boole axioms for the logical conjunction are:

$$\begin{array}{l} a\wedge \left(b\wedge c\right)=\left(a\wedge b\right)\wedge c\text{ Associativity}\\ a\wedge b=b\wedge a\text{ Commutativity}\\ a\wedge \left(a\vee b\right)=a\text{ Absorption}\\ a\wedge \left(b\vee c\right)=\left(a\wedge b\right)\vee \left(a\wedge c\right)\text{ Distributivity}\\ a\wedge \neg \;a=\emptyset \text{ Complement} \end{array}$$

In addition, the De Morgan laws relating the negation of composed Boolean propositions

¬(a∧b)=¬ a∨¬ b;  ¬(a∨b)=¬ a∧¬ b

allow to define the corresponding axioms for the logical disjunction. The consideration of these rules allow the simplification of complex propositions involved in the analysis of Boolean networks and may be useful even in cases where a relatively large number of nodes is involved, as in the 46-node network introduced in this work. For example, use of the absorption property in the expression

SOS=CD28∨(CD28∧CD25∧¬ CTLA4dimers)=CD28

leads to the conclusion that only CD28 is actually relevant for the activation of SOS, in spite of the presence of other network components in the interactive rule. The Boolean rules are also consistent with the collapse of linear strings of the network into a single node. The recursive use of the Boolean algebraic methods conduced to a final set of analytic propositions, presented in Tables 1, 2 in the text, which define the steady-state expression of all the network components. All of these are specified only by the state of the input nodes: TCR, CD80/86, and AMPK. Similarly, the results arising in an intermediate stage of the algebraic evaluation conduced to the 12-node simplified rules involved in the analysis of the attraction basins presented in Figure 7.
