# Supplementary material for: An Integrative Network Modeling Approach to T CD4 Cell Activation
Source: Front Physiol. 2020 Apr 23;11:380. doi: 10.3389/fphys.2020.00380 (PMC7212416; doi:10.3389/fphys.2020.00380)
Supplement: Supplementary file 1 [file Data_Sheet_1.PDF]

Supplementary Table 1

| Node | Function |
|---|---|
| **TCR** | TCR & ! CTLA4dimers |
| **CD28** | CD8086e & ! CTLA4dimers |
| **CD8086** | CD8086e & ! CTLA4dimers |
| **AP1** | rasGTPr |
| **CD25** | IL2G & ! CTLA4dimers |
| **IL2G** | NFAT & AP1 & ! NDRG1 & ! RORGT & ! IL21e & ! IL21\| NFKB & NFAT & AP1 & ! NDRG1 & ! RORGT & ! IL21e & ! IL21 \| STAT5 & NFAT & AP1 & ! NDRG1 & ! RORGT & ! IL21e & ! IL21 |
| **MTOR** | CD25 \| AKT |
| **ZAP70** | (TCR & LCK) & ! CTLA4dimers |
| **STAT5** | CD25 & ! CTLA4dimers |
| **NFAT** | CA \| CA & AKT |
| **NFKB** | (PKC) \| (PKC & PDK1) |
| **AKT** | (CD28 & ! CTLA4dimers) \| PDK1 |
| **CTLA4** | (IL2G & ZAP70) |
| **CTLA4dimers** | CTLA4 & CD8086l \| FOXP3 & TGFB |
| **BCL2** | AKT |
| **NDRG1** | NFAT & ! AKT |
| **DAG** | (PLC) \| (AKT & PLC) |
| **SOS** | CD28 \| CD28 & CD25 |
| **RasGTPr** | LAT & SOS & DAG |
| **LCK** | TCR & ! CTLA4dimers |
| **PDK1** | CD25 \| CD28 |
| **MTORC1** | MTOR & ! AMPK |
| **AMPK** | AMPK |
| **MTORC2** | MTOR & AMPK |
| **LAT** | ZAP70 |
| **PLC** | ZAP70 |
| **PIP2** | PLC |
| **PIP3** | PIP2 |
| **CA** | PIP3 |
| **PKC** | DAG |
| **TBET** | (MTORC1 & IL12e & IFNGe & NFKB & NFAT & AP1) & !IL4 & !IL10 & !GATA3 |
| **IFNG** | (TBET & AP1 & NFAT) |
| **IFNGe** | IFNGe |
| **IL12e** | IL12e |
| **GATA3** | ((MTORC2 & IL4e & STAT5 & NFAT) & (!TBET & !TGFB & !IFNG) |
| **IL4** | (GATA3 & !TBET) & !IFNG |
| **IL4e** | IL4e |
| **FOXP3** | ((NFAT & STAT5 & AP1 & IL2G & TGFBe & IL10e) \| (IL10 & TGFBe & IL10e & CTLA4) \| (TGFB & TGFBe)) & ! IFNG |
| **IL10** | FOXP3 & TGFBe |
| **TGFB** | FOXP3 |
| **TGFBe** | TGFBe |
| **IL10e** | IL10e |
| **RORGT** | (CD28 & MTORC1 & IL21e & TGFBe) & ! (TBET \| FOXP3 \| GATA3) |
| **IL21** | ( IL21e \| RORGT ) & ! (IFNG \| IL4 \| IL10 ) |
| **IL17** | RORGT |
| **IL21e** | IL21e |

Mathematical notation: ! for NOT, | for OR, and & for AND.
