# Supplementary material for: An Integrative Network Modeling Approach to T CD4 Cell Activation
Source: Front Physiol. 2020 Apr 23;11:380. doi: 10.3389/fphys.2020.00380 (PMC7212416; doi:10.3389/fphys.2020.00380)
Supplement: Supplementary file 2 [file Data_Sheet_2.PDF]

Supplementary Table 2

Nodes	Interactions (downstream)			
	Stimulatory	References	Inhibitory	References
TCR	ZAP70, LCK	Stirnweiss et al. 2013, Gaud et al. 2018		
LCK	ZAP70	Stirnweiss et al. 2013, Gaud et al. 2018		
ZAP70	LAT, CTLA4	Gaud et al. 2018		
LAT	PLC, RASGTP	Gaud et al. 2018		
CD8086	CD28	Vandenborre et al. 1999, Khailaie et al. 2018	CTLA4dimers	Schneider et al 2008, Darlington et al. 2005
PDK1	AKT, NFKB	Chen et al. 2013		
RasGTP	AP1	Gaud et al. 2018		
DAG	PKC, RASGTP	Janardhan et al. 2011, Mor et al. 2007, Macian et al. 2002, Su et al. 1994, PerezdeCastro et al. 2004, Gaud et al. 2018		
PLC	PIP2, DAG	Gaud et al. 2018		
AKT	DAG, BLC2, NFAT, MTOR	Janardhan et al. 2011, Mor et al. 2007, Macian et al. 2002, Su et al. 1994, PerezdeCastro et al. 2004, Gaud et al. 2018, Jung et al. 2018, Frauwirth et al. 2002, So et al. 2013	NDRG1	Oh et al. 2015
CA	NFAT	Chen et al. 2013		
PKC	NFKB	Gaud et al. 2018		
PIP2	IP3	Gaud et al. 2018		
IP3	CA	Chen et al. 2013		
CD28	PDK1, SOS, AKT	Chen et al. 2013, Anardhan et al. 2011, Mor et al. 2007, Macian et al. 2002, Su et al. 1994, PerezdeCastro et al. 2004		
CTLA	FOXP3, CTLA4dimers	Vandenborre et al. 1999, Khailaie et al. 2018, Wing et al. 2008, Walker et al. 2013		
CTLA-4dimers			CD8086, LCK AKT, STAT5, ZAP70, CD25, CD28, TCR	Chikuma et al. 2017, Vandenborre et al. 1999, Khailaie et al. 2018
CD25	PDK1, SOS, STAT5, MTOR	Park et al. 2009, Gadina et al. 1999, Marzec et al. 2008, Mahmud et al. 2013		
STAT5	FOXP3, GATA3, IL2G	Waters et al. 2018, Mahmud et al. 2013		
NDRG1			RORGT, IL2G	Oh et al. 2015
BCL2				
SOS	RASGTP	Oh et al. 2015		
MTOR	MTORC1, MTORC2, GATA3	Shi et al. 2011, Dang et al. 2011		
MTORC1	RORGT, TBET			
MTORC2	GATA3	Michalek et al. 2011, Delgoffe et al. 2011,Klysz et al. 2015		
AMPK	MTORC1, MTORC2	Michalek et al. 2011, Delgoffe et al. 2011,Klysz et al. 2015, MacIver et al. 2013, Man et al. 2015		
IL2G	FOXP3, CTLA4, CD25	Perez et al. 1997, Wang et al. 2001		
AP1	FOXP3, INFG,TBET, IL2G	Sereti et al. 2000, Waters et al. 2018, MacIver et al. 2013, Ichiyama et al. 2008, Wan et al. 2014		
NFKB	TBET, IL2G	Oh et al. 2015, MacIver et al. 2013, Ichiyama et al. 2008, Wan et al. 2014		
NFAT	FOXP3, GATA3, IFNG, TBET, NDRG1, IL2	Sereti et al. 2000, Waters et al. 2018, MacIver et al. 2013, Ichiyama et al. 2008, Wan et al. 2014		
GATA3	IL4	Chen et al. 2013, Hori et al. 2003, Davidson et al. 2007, Zheng et al. 2007	TBET, RORGT	MacIver et al. 2013,Ichiyama et al. 2008,Wan et al. 2014
FOXP3	TGFB, IL10, CTLA4dimers	Wing et al. 2008,Walker et al. 2013, MacIver et al. 2013, Ichiyama et al. 2008, Wan et al. 2014	RORGT	MacIver et al. 2013,Ichiyama et al. 2008,Wan et al. 2014
RORGT	IL17, IL21	Chen et al. 2013, Hori et al. 2003, Davidson et al. 2007, Zheng et al. 2007	IL2G	Chen et al. 2013,Hori et al. 2003,Davidson et al. 2007,Zheng et al.
TBET	INFG	MacIver et al. 2013,Ichiyama et al. 2008,Wan et al. 2014	GATA3, IL4, RORGT	Chen et al. 2013,Hori et al. 2003,Davidson et al. 2007,Zheng et al.
IL4e	GATA3	Chen et al. 2013, Hori et al. 2003, Davidson et al. 2007, Zheng et al. 2007		
IL12E	TBET	Chen et al. 2013, Hori et al. 2003, Davidson et al. 2007, Zheng et al. 2007		
IFNGe	TBET	MacIver et al. 2013,Ichiyama et al. 2008,Wan et al. 2014		
IL10e	FOXP3	MacIver et al. 2013,Ichiyama et al. 2008,Wan et al. 2014		
TGFBe	RORGT, FOXP3	Chen et al. 2013, Hori et al. 2003, Davidson et al. 2007, Zheng et al. 2007		
IL21e	IL21, RORGT	Chen et al. 2013, Hori et al. 2003, Davidson et al. 2007, Zheng et al. 2007	IL2G	Chen et al. 2013,Hori et al. 2003,Davidson et al. 2007,Zheng et al.
IL4			IL21, TBET	Chen et al. 2013,Hori et al. 2003,Davidson et al. 2007,Zheng et al.
IL10			IL21, TBET	Chen et al. 2013,Hori et al. 2003,Davidson et al. 2007,Zheng et al.
TGFB	CTLA4dimers	Chen et al. 2013, Hori et al. 2003, Davidson et al. 2007, Zheng et al. 2007	GATA3	MacIver et al. 2013,Ichiyama et al. 2008,Wan et al. 2014
IL21	IL2G	Chen et al. 2013, Hori et al. 2003, Davidson et al. 2007, Zheng et al. 2007		
IL17				
IFNG			IL21, IL4, FOXP3, GATA3	MacIver et al. 2013,Ichiyama et al. 2008,Wan et al. 2014
